# Supplementary material for: Self-Powered Flexible Sour Sensor for Detecting Ascorbic Acid Concentration Based on Triboelectrification/Enzymatic-Reaction Coupling Effect
Source: Sensors (Basel). 2021 Jan 7;21(2):373. doi: 10.3390/s21020373 (PMC7827105; doi:10.3390/s21020373)
Supplement: Supplementary file 1 [file sensors-21-00373-s001.zip › SI.docx]

**Supplement material**

Self-powered flexible sour sensor for detecting ascorbic acid concentration based on triboelectrification/enzymatic-reaction coupling effect

Tianming Zhao^1^, Qi Wang^1,*^ and An Du^1,*^.

^1^ College of Sciences, Northeastern University, Shenyang 110819, People’s Republic of China; zhaotm@stumail.neu.edu.cn

***** Correspondence: wangqi@mail.neu.edu.cn; du_an_neu@126.com


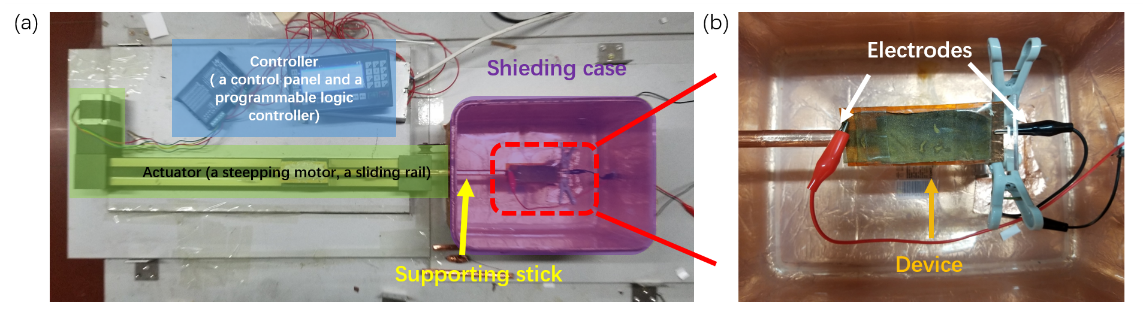


**Figure S1**. The photograph of the measurement system (a) and the detail of device (b). When the measurement system works, the device will be deformed by the supporting stick and output triboelectric current.

**
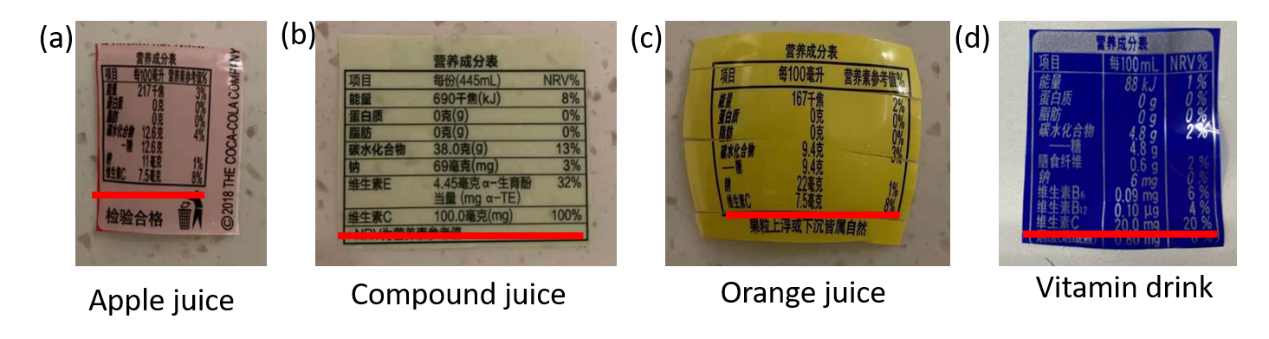
**

**Figure S2**. The AA concentration of apple juice (a), compound juice (b), orange juice (c) and vitamin drink (d).
