# Supplementary figures and images for: Self-Powered Flexible Sour Sensor for Detecting Ascorbic Acid Concentration Based on Triboelectrification/Enzymatic-Reaction Coupling Effect
Source: Sensors (Basel). 2021 Jan 7;21(2):373. doi: 10.3390/s21020373 (PMC7827105; doi:10.3390/s21020373)

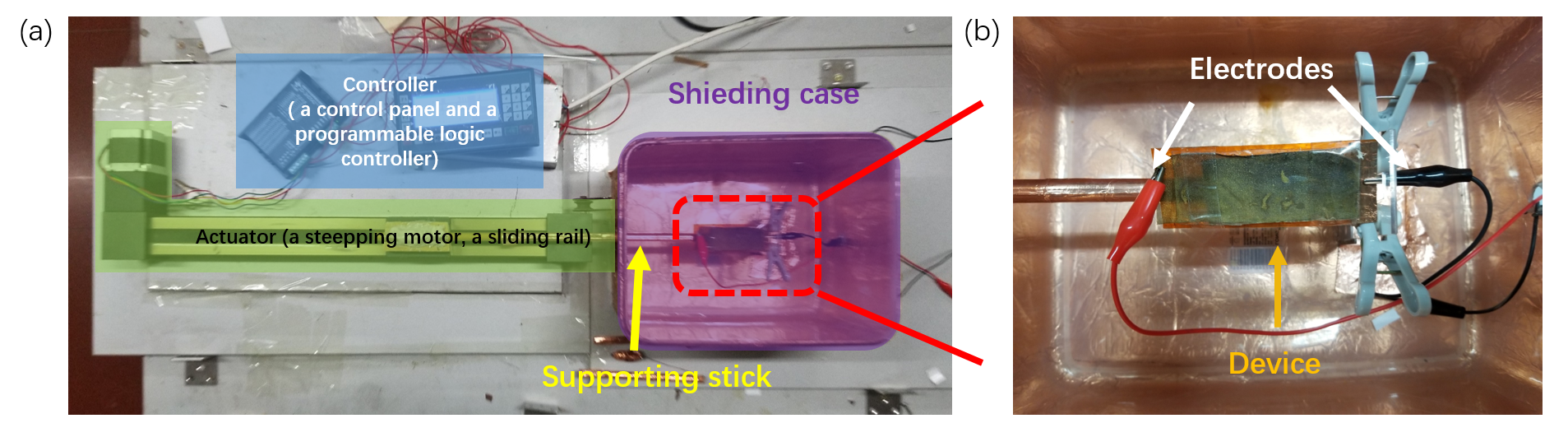

Supplement: Supplementary file 1 [file sensors-21-00373-s001.zip › FigureS1.png]

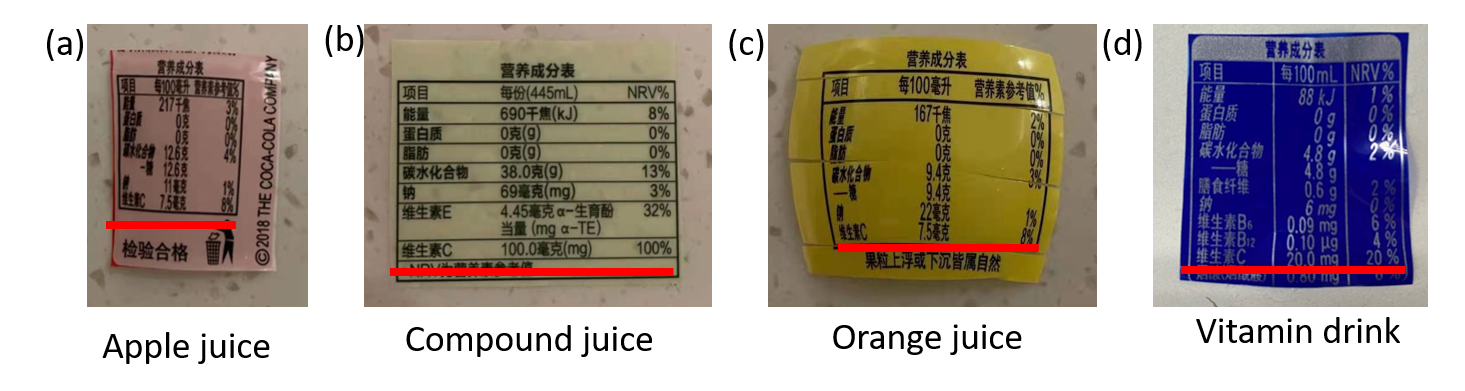

Supplement: Supplementary file 1 [file sensors-21-00373-s001.zip › FigureS2.png]
